# Is there an agreement between self-reported medical diagnosis in the CARTaGENE cohort and the Québec administrative health databases?

**DOI:** 10.23889/ijpds.v5i1.1155

**Published:** 2020-03-26

**Authors:** Y Payette, CS de Moura, C Boileau, S Bernatsky, N Noisel

**Affiliations:** 1 CARTaGENE Cohort and Biobank, CHU Sainte-Justine, Montréal, Québec, Canada; 2 Department of Epidemiology, Biostatistics and Occupational Health, McGill University, Montréal, Québec, Canada; 3 Division of Clinical Epidemiology, McGill University Health Centre, Montréal, Québec, Canada; 4 Department of Environmental and Occupational Health, School of Public Health, University of Montreal, Montreal, Québec, Canada

## Abstract

**Background:**

Population health studies often use existing databases that are not necessarily constituted for research purposes. The question arises as to whether different data sources such as in administrative health data (AHD) and self-report questionnaires are equivalent and lead to similar information.

**Objectives:**

The main objective of this study was to assess the level of agreement between self-reported medical conditions and medical diagnosis captured in AHD. A secondary objective was to identify predictors of agreement among medical conditions between the two data sources. Therefore, the purposes of the study were to explore the extent to which these two methods of commonly used public health data collection provide concordant records and identify the main predictors of statistical variations.

**Methods:**

Data were extracted from CARTaGENE, a population-based cohort in Québec, Canada, which was linked to the provincial health insurance records of the same individuals, namely the MED-ÉCHO database from the *Régie de l’assurance maladie du Québec* (RAMQ) and the fee-for-service billing records provided by the physician, for the time period 1998-2012. Agreement statistics (kappa coefficient) along with sensitivity, specificity and predictive positive value were calculated for 19 chronic conditions and 12 types of cancers. Logistic regressions were used to identify predictors of concordance between self-report and AHD from significant covariates (sex, age groups, education, region, income, heavy utilization of health care system and Charlson comorbidity index).

**Results:**

Agreement between self-reported data and AHD across diseases ranged from kappa of 0.09 for chronic renal failure to 0.86 for type 2 diabetes. Sensitivity of self-reported data was higher than 50% for 14 out of the 31 medical conditions studied, especially for myocardial infarction (88.62%), breast cancer (86.28%), and diabetes (85.06%). Specificity was generally high with a minimum value of 89.70%. Lower concordance between data sources was observed for higher frequency of health care utilization and higher comorbidity scores.

**Conclusion:**

Overall, there was moderate agreement between the two data sources but important variations were found depending on the type of disease. This suggests that CARTaGENE’s participants were generally able to correctly identify the kind of diseases they suffer from, with some exceptions. These results may help researchers choose adequate data sources according to specific study objectives. These results also suggest that Québec’s AHD seem to underestimate the prevalence of some chronic conditions, which might result in inaccurate estimates of morbidity with consequences for public health surveillance

## Introduction

In epidemiological and research studies, self-reported questionnaires are commonly used to obtain information on health status, prevalence of chronic conditions, and medication use [1, 2]. However, the reliability of self-reported medical conditions has been shown to vary according to sociodemographic subgroup and/or comorbidity status [11-6]. The accuracy of self-reported questionnaires may be affected by several factors, including the respondent’s ability to recall or fully understand the diagnosis, or his/her willingness to disclose medical information, or the complexity of the diagnosis itself [7, 8].

Medical records derived from administrative health data (AHD) are often used to validate self-reported data [9-11]. While AHD is not primarily collected for disease surveillance or research purposes, it remains an essential source of data for public health agencies throughout Canada to monitor statistics such as prevalence, incidence and temporal trends [4, 9, 12-14]. For example, in Canada, the *Canadian Chronic Disease Surveillance System* (CCDSS) is a collaborative network of provincial and territorial chronic disease surveillance systems, led by the Public Health Agency of Canada (PHAC). Its aim is to foster the collection of surveillance data in a consistent and comparable way across all provinces and territories [15-18]. In Québec, the *Système intégré de surveillance des maladies chroniques du Québec* (SISMACQ) is based on five administrative health databases and Public Health institutions for surveillance purposes [19].

Previous studies have shown greater agreement between AHD and medical records than between AHD and patient self-reported data [2, 3, 13, 20]. Yet AHD is not error-proof and updates in classification codes can lead to errors [19, 21-26].

The main objective of this study is to assess the level of agreement between CARTaGENE’s self-reported disease and medical diagnoses captured in the AHD by measuring the kappa, sensitivity, specificity and positive predictive value (PPV). In addition, this study aims to identify the main predictors of agreement between these two types of data sources.

## Methods

### Data sources

#### The CARTaGENE baseline health survey

CARTaGENE is a public health research platform created for the investigation of the risk factors of health and diseases in an aging population in Québec, Canada. CARTaGENE’s mission is to accelerate health research and lower associated costs, and to support evidence-based decision making in clinical practice and public health. Consisting of a rich collection of data including phenotyping and genotyping data, CARTaGENE is the largest ongoing prospective population cohort in Québec, Canada, and a biobank of 43,000 participants [27].

Our analysis focuses on the first CARTaGENE wave, which comprises data for participants recruited from July 2009 to October 2010. Details about recruitment and sample selection have been described previously [27]. Briefly, participants were randomly selected to be broadly representative of the population recorded on provincial health insurance registries - *FIPA* files (*Fichier administratif des Inscriptions des Personnes Assurées de la Régie d’Assurance Maladie du Québec* (RAMQ)) in metropolitan areas. The random selection was based on the survey design which included two age groups (40-54 and 55-69 years-old), sex and forward sortation area (defined by the first three characters in a Canadian postal code) to reflect the population density from the 2006 Census. Probability proportional to size was used to define quotas for each of these strata. Participants were between 40 and 69 years old and came from four metropolitan areas in the province (Montréal, Québec, Sherbrooke and Saguenay). A total of 19,996 men and women enrolled in the study, representing 1% of the Québec urban population. Proportions of women and men (51.6% vs. 48.4%) were similar to the 2006 Census data for the same population subgroup [27]. Most of them were born in Canada (83.5%) and spoke French (78.6%). A high proportion of participants were married (63.7%) and employed (65.5%).

The recruitment of participants was achieved through a call centre at the RAMQ. Information packages were first sent by mail. Potential participants were subsequently contacted by telephone and those interested were scheduled for an interview in one of the 12 clinical assessment sites. During the visit at clinical sites, participants signed a consent form [28] and filled out questionnaires: a Computer-Assisted Personal Interview (CAPI) for self-reported socio-demographic factors and lifestyle, and a questionnaire administered by a nurse or interviewer for medical conditions. Participants were also asked to provide biosamples (blood and urine) and physical measurements (e.g., blood pressure) under strict Standard Operating Procedures (SOPs).

CARTaGENE questionnaires included questions about different topics such as socio-demographic factors, lifestyle, mental status, psychosocial environment, individual and family history of disease, medical care system and medication intake. All the questionnaires were developed, validated and used in other large-scale surveys such as the *Canadian Health Measures Survey* (CHMS), the *International Physical Activity Questionnaire* (IPAQ [29]) or came from clinical tools commonly used, like the General Anxiety Disorder-7 (GAD-7) and Patient Health Questionnaire (PHQ-9) to assess mental health. The self-reported disease diagnoses were retrieved from the section regarding the individual history of disease which uses the same questions as the *US National Health and Nutrition Examination Survey* (NHANES, wwwn.cdc.gov) [31]. The section covered questions on more than 30 medical conditions, including chronic diseases and cancers. To allow capturing of self-reported conditions, all questions in this section used a similar wording structure: “Has a doctor ever told you that you had … (name of the disease)” (see Appendix A). 

#### The MED-ÉCHO administrative health data (AHD)

As part of the informed consent process [28], CARTaGENE participants agreed to the linkage of their questionnaires with governmental health databases. The MED-ÉCHO AHD contains RAMQ diagnoses, patient demographics, hospital admissions, physician claims, and discharge dates of all Québec residents encoded by the International Classification of Diseases, Ninth or Tenth Revision (ICD-9, ICD-10) [32]. Access to AHD was granted from 1998 (no data available prior to this date) to one year after the participant’s consent date for every CARTaGENE participant (consents given between 2009-07-29 and 2011-03-01). The RAMQ used the participant’s encrypted health insurance number to link AHD to CARTaGENE data. It was evaluated that 95% of participants of the cohort had a least one non-ICD code “V999” in their AHD, indicating uncategorized diseases.

### Data treatment

#### Determination of self-reported and AHD disease diagnosis

The selection of medical conditions for the study was based on two main criteria: 1) the availability of the data in both the CARTaGENE database and AHD availability for the same medical conditions, and 2) the relevance for disease surveillance or the burden of disease for public health prevention. This includes all chronic diseases with high prevalence or high disability adjusted life years (DALY) [33] as well as cancers. The list of selected medical conditions and the corresponding ICD codes used to AHD extraction for cases identification are presented in Appendix A. We excluded rare conditions or orphan diseases from the analysis as the low number of occurrences did not allow statistical analysis with sufficient statistical power. In order to capture the same diseases and conditions in the AHD records as in self-reported data, we relied on pre-existing validated algorithms that allow identification of their occurrence using inpatient and outpatient claims data within a time range [34, 35] (Appendix A). For instance, a validated case of asthma is a positive answer to the question “Has a doctor ever told you that you had asthma?” (CARTaGENE self-report data) or one hospitalization or three physician claims in two years or less involving the codes 493 (ICD-9) or J45 (ICD-10). Quan’s coding was used to capture the comorbidity status as defined by the Charlson comorbidities Index using the AHD [34-36]. 

#### Agreement estimates

The overall frequencies of medical conditions found in self-reported diagnosis data were compared to the frequencies of medical conditions retrieved using the MED-ÉCHO AHD. Binary classes of concordant cases (category self-reported positive / AHD positive and category of self-reported negative / AHD negative being positive concordant cases) and discordant cases (AHD positive with CARTaGENE negative, or vice-versa, being negative discordant cases) were created. Then, using the MED-ÉCHO AHD as gold standard, Cohen’s kappa coefficient (inter-rater agreement taking into account the possibility of the agreement occurring by chance), sensitivity (proportion of positives that are correctly identified), specificity (proportion of negatives that are correctly identified), and positive predictive value (PPV - proportions of true positives over positive calls) were determined for each disease and cancer included in the study. The kappa values, which result from a combined frequency analysis of looking at both sensitivity and specificity [37], were considered as follows: below 0.40 was considered poor-to-fair agreement, 0.41 to 0.60 was moderate agreement, 0.61 to 0.80 was good agreement, 0.81 to 1.00 was excellent agreement [38].

### Factors associated with agreement

Specific social determinants of health (age groups, sex, region of residence (Montréal, Québec, Sherbrooke and Saguenay), education (high school or less, college, and university or higher) and income (expressed as quintiles: missing information was included in a single missing category (6.6% of income data)), that are generally found in the epidemiological literature were included as covariates for their possible influence on concordance [39]. Comorbidities have been reported to affect the agreement between self-report and AHD medical conditions mainly by introducing confusion in the participant’s recall. In this study, the participant’s comorbidity was considered as a relevant covariate and assessed by calculating the Charlson comorbidity index (CCI) using AHD [40, 41]. Heavy utilization of health care system was defined as 20 or more physician or hospital claims found in a three year time period prior to the participants’ recruitment [42-44] also using AHD [35].

### Statistical analysis

To identify how selected predictors influenced the concordance, logistic regression analyses (odd ratios) were performed for each medical condition where the number of concordant cases from the two data sources (positive self-report in CARTaGENE and positive AHD) were greater than 30. Covariates for the full logistic models included age groups, sex, region of residence, education, income, heavy utilization of health care and the CCI [2, 41, 45]. Since we wanted to compare the importance of the covariates using the same model for all selected medical conditions, no partial models or interaction terms were investigated. For the predictors identified as significant covariates, odds ratios (OR) along with confidence interval (CI) were calculated based on logistic regressions. Analyses were performed using SAS Version 9.4 (2004; SAS Institute Inc., Cary, NC) for model statistics (Log-likelihood, Wald probabilities) and were considered statistically significant for values of p <0.05.

## Results

### Population characteristics

Of the 19,996 CARTaGENE participants, 10,310 were women (52%) (Table 1). The median participant’s age was 53 ± 7.9 years. Most participants were recruited in the Montreal area (76%), and 72% had a college education or higher. Only 4.5% of participants had CCI equal or greater than three, and 56% of them were heavy health care system users.

### Agreement

The three most frequent conditions in both AHD and CARTaGENE self-reported data, respectively, had small differences in frequencies: hypertension (25.0% and 22.8%), osteoarthritis (16.1 and 20.5%) and depression (16.7 and 18.4%). Frequencies were generally slightly higher in AHD for 13 out of 19 diseases (68%) and nine out of twelve cancer types (75%) (Table 2). Conversely, hypertension, asthma, rheumatoid conditions including rheumatoid arthritis, irritable bowel syndrome and myocardial infarction were reported more frequently in the CARTaGENE self-reported data than in AHD.

Good to excellent agreements (kappa > 0.61) were found in hypertension, diabetes, hypothyroidism, myocardial infarction, multiple sclerosis, and in breast, prostate, thyroid, bladder, lung and kidney cancers. Therefore, five out of 19 chronic diseases (26%) had good to excellent kappa whereas for the cancers, a high kappa coefficient was more frequently observed (six out of twelve cancers, 50%). The kappa statistic was moderate (0.41 to 0.60) for depression, asthma, stroke, Crohn’s disease, schizophrenia, epilepsy, Parkinson’s disease, as well as for colon cancer and non-Hodgkin’s lymphoma. Agreement was poor (kappa < 0.40) for chronic obstructive pulmonary disease, chronic renal failure, irritable bowel syndrome, rheumatoid arthritis, osteoarthritis and systemic lupus erythematosus, as well as for melanoma, cervical, rectum and uterine cancers.

There were 14 out of 31 diseases/cancers (45%) with sensitivity higher than 50%, including among the highest: myocardial infarction (88.62%), breast cancer (86.28%), and diabetes (85.06%). Chronic renal failure had the lowest sensitivity (5.09%). Sixteen other conditions had low sensitivity (<50%), including neurological disorders (Parkinson’s disease and schizophrenia), diseases of the digestive system (irritable bowel syndrome and Crohn's disease), and several types of rare cancers (lung, uterine, melanoma, colon, non-Hodgkin’s lymphoma, cervical, and rectum). The specificity values for all the conditions were above 90%, except for osteoarthritis (89.7%). PPV ranged from 16.03% (cervical cancer) to 100% (Parkinson’s disease), with most diseases (21/32) having a PPV greater than 50%. 

### Covariates affecting agreement

The logistic model statistics (Table 3) shows which covariates affected the concordance in this study. Variation of concordance was often associated with sex (Fig 1A). For seven medical conditions (chronic obstructive pulmonary disease, osteoarthritis, asthma, irritable bowel syndrome, depression, hypothyroid, systemic lupus erythematosus, and breast cancer), women were less prone than men to report diagnoses concordant with AHD. However, the opposite was observed for ten other medical conditions, including myocardial infarction and chronic renal failure. Furthermore, sex had no impact on agreement for nine other medical conditions. For all medical conditions, except for cervical, uterine, kidney and bladder cancers, being a heavy health care user was associated with a lower likelihood of having concordance between the two data sources for a light health care user (Fig 1B). In some cases, this effect was two-fold or more (e.g., schizophrenia). Increase of CCI was associated with a lower probability of agreement for all medical conditions except for schizophrenia, multiple sclerosis, osteoarthritis, irritable bowel syndrome and Parkinson’s disease (Fig 1C). Variations in concordance were also observed between age groups. Specifically, when comparing age groups 60-69 years-old to 40-49 years-old for schizophrenia, multiple sclerosis, asthma, irritable bowel syndrome, and depression, the older age group had more concordant cases compared to the younger age group (Fig 1D). For chronic renal failure, hypothyroid, hypertension, rheumatoid arthritis, diabetes, melanoma cancer, osteoarthritis, stroke, myocardial infarction and prostate cancer, the older age group was less concordant compared to the younger age group (Fig 1D).

Income had almost no statistical effect on agreement (Table 3 and Fig 1E). However, even with large confidence intervals, participants with the highest income were more likely to report concordant information than participants with the lowest income. One exception was melanoma cancer, where the highest income was associated with lower agreement. 

## Discussion

### Main results

In this study, we assessed the agreement between self-reported diagnosis and AHD for 19 diseases and twelve cancers in a large ongoing prospective study (CARTaGENE). Overall, there was good agreement between the two data sources for specific diseases such as diabetes; other diseases showed moderate to poor agreement (i.e., chronic renal failure or cervical cancer). Similar findings, i.e. variations of kappa or PPV across diseases were also observed in previous studies [46-49].

In general, good to excellent kappa coefficients (inter-rater agreement) were found more often for cancers than for other chronic diseases. Specificity remained relatively high for all chronic diseases and cancers; revealing that, generally speaking, absence of diseases and cancers was correctly reported by the majority of this study population. Interestingly, the two most prevalent cancers, breast and prostate, presented with sensitivity and PPV values among the highest for cancers. This can be explained by a strong agreement and by the influence of prevalence on PPV.

Some studies have shown that demographic factors, such as sex and age can affect self-reporting of diseases [22, 48, 50]. This bias does not occur in AHD. Nevertheless, self-report questionnaires can be an important source of clinical information for both epidemiological research and population surveillance [35, 50-54]. In this study, the demographic profile of the people for whom self-reported data is concordant with AHD seemed to vary by disease. In most diseases studied here, age groups, heavy health care utilization, comorbidity and in some instances, higher income, had an influence in the level of concordance. We did not observe the same agreement trend of demographic factors among all chronic diseases; this may be explained by different biases, specific to each disease. For example, a previous study on chronic renal failure reported that only 8% of the CARTaGENE participants were aware of their chronic kidney condition [55]. Our results align with these previous findings given that the sensitivity for chronic renal failure was 5.1% in our study. Sensitivity was lowest for chronic diseases or cancers that could have been more difficult to diagnose for physicians or to remember for participants [55], depending on specific bias of each medical condition. For cancers, the high level of agreement observed may be related to the recollection of diagnosis by the participant. Most often, cancers can be more specifically described by physicians and easily remembered by participants, especially when hospitalization is needed. Additionally, diagnostic procedures and intensive treatments may play a key role in the participants’ recall for severe diagnosis [56]. 

### Limitations

There are important limitations and bias in both data sources. In general, MED-ÉCHO AHD is used in Québec for the population surveillance of prevalence and incidence of diseases [57] and is considered as the gold standard. Some diseases diagnosed in a clinical setting may not be correctly coded in the MED-ÉCHO AHD; a physician can fill out a claim to the RAMQ for a consultation without specifying any ICD code (in Québec this is coded as “V999” and is not part of the official ICD coding). This is likely to affect chronic conditions such as depression, diabetes, hypertension and osteoarthritis; but would rarely affect cancers. Further analysis of the “V999” coding per hospital/physician’s practice is needed to clarify this phenomenon.

Another major limitation may be due to MED-ÉCHO AHD being available only after 1998. Due to this limitation, some diseases may have been self-reported by the participant but not captured in the AHD. However, in 1998, the oldest participant of this study was about 57 years old, and most of the participants were below 40 years old. Since the onset of most studied diseases is generally over 40 years of age, this time limitation would probably have a slight impact on agreement, except for diseases usually appearing earlier in life. Indeed, this is the case for schizophrenia and multiple sclerosis, which showed low sensitivity (respectively 35.37% and 56.49%). As for asthma, some studies reported that 42% of adults with active asthma had disease onset before age 16 [58], which might have explained the observed discrepancy in agreement for this disease (kappa = 0.47).

In self-reported questionnaires, interviewees can be biased by the way they understand a question and their recollection of the diagnosis, or their understanding can be affected by other bias [59]. For example, some confusion may arise from similar terms (osteoarthritis versus rheumatoid arthritis), or similar cancer location (cervical cancer versus uterine cancer), which might explain the low concordance. Investigating specific covariates and patterns for each disease may improve the understanding of factors underlying these discrepancies.

The use of hospital-based or physician records could have helped to capture more precisely the medical conditions for the studied population. However, this was not possible since the CARTaGENE consent does not allow the access to these records. For this reason, only AHD could be used for this study, including all the inherent limitations (limited time- period and coding issues for example).

More in-depth research on each specific medical conditions and diagnosis is needed in order to better understand the determinants of agreement between the two data sources. However, even if some specific hypotheses are addressed here, the purpose of this study was not to describe all bias that might affect the agreements of selected diseases or cancers, but to describe how self-declared information and AHD could be related to one another in a specific cohort setting.

### Strengths of the study

Scientific literature gathers numerous studies analyzing the agreement between two data sources, generally self-reported and AHD. However, these studies mainly focus on only one (or two) specific diseases and/or diagnosis [13, 22, 50]. Very few studies include a broad range of medical conditions (including cancers) to access agreement between two data sources. In a recent publication, self-reporting of chronic conditions seemed to underestimate the prevalence of many chronic conditions in Québec, thus resulting in less accurate estimates of multimorbidity, such as in our study [49].

For the self-declared diseases, observed discrepancies in concordance is unlikely to arise from the questionnaire wording, as the questions are always the same for all medical conditions of interest. This consistency of wording avoids random error.

The high level of agreement observed for cancers is probably related to the recollection of diagnosis by the participant and the fact that, most often, Physicians can describe cancers more specifically and they are more easily remembered by participants, especially when hospitalization is needed. Additionally, diagnostic procedures and intensive treatments may play a key role in the participants’ recall for such a severe diagnosis.

This study also has meaning in the context of population surveillance based on the MED-ÉCHO AHD. Even if good agreement was found for some diseases and cancer, small differences in disease frequencies might have an impact on population surveillance by health agencies in Canada. As an example, for hypertension, a difference of 2% at the population level (25.0% versus 22.8% in this study) represents more than 70,000 persons among the 40-70 years-old category having hypertension in Québec than evaluated using the AHD as the gold standard. The burden of hypertension was evaluated as being of 1,300,000 people in the Québec population above 20 years-old for the 2006-2007 period [60]. Hypertension has a significant impact on cardiovascular disease and its under-evaluation might reveal unsuspected public health issues, such as those identified in chronic kidney disease [55]. 

## Conclusion

Large population-based cohorts are useful tools in epidemiology, public health and genetic studies. Prospective continuous medical self-reporting data in population-based cohorts, like in the CARTaGENE cohort, is therefore of great importance for research, such as monitoring agreement with AHD

## Acknowledgments

The authors would like to thank all the CARTaGENE participants for their generous investments in health research. The authors would also like to thank the *Régie de l’assurance maladie du Québec* (RAMQ) and the *Commission d’accès à l’information* (CAI) for their support in obtaining the data relevant to the study.

### Ethics approval

CARTaGENE has obtained ethics approval from the CHU Sainte-Justine under the reference: MP-21-2011-345, 3297. The latest annual ethics renewal was granted on September 13, 2019. 

## Supplementary Material

Tables and Figures
