# Supplementary material for: Is there an agreement between self-reported medical diagnosis in the CARTaGENE cohort and the Québec administrative health databases?
Source: Int J Popul Data Sci. 2020 Mar 26;5(1):1155. doi: 10.23889/ijpds.v5i1.1155 (PMC7473265; doi:10.23889/ijpds.v5i1.1155)
Supplement: Tables and Figures [file ijpds-05-1155-s001.pdf]

Table 1. Characteristics of participants (N=19,996), first CARTaGENE wave (2009-2010)

Characteristics	N (%)
Sex	
Men	9,686 (48.4%)
Women	10,310 (51.6%)
Age	
40-49	6,935 (34.7%)
50-59	7,602 (38.0%)
60-69	5,459 (27.3%)
Region	
Montréal	15,227 (76.2%)
Saguenay	799 (4.0%)
Québec city	3,045 (15.2%)
Sherbrooke	925 (4.6%)
Education	
High school or less	5,185 (25.9)
College/vocational training	6,325 (31.6%)
University or more	8,357 (41.8%)
Missing	129 (0.6%)
Income quintiles	
Lowest (24999$ and lesss)	2,465 (12.3%)
Low-middle (25 000$ to 49 999$)	4,361 (21.8%)
Middle (50 000$ to 74 999$)	4,057 (20.3%)
Upper-middle (75 000$ to 99 999$)	5,831 (29.2%)
Highest (100 000$ and up)	1,961 (9.8%)
Missing	1,321 (6.6%)
Health care utilization	
< 20 claims in last 3 years	8,837 (44.2%)
≥ 20 claims in last 3 years	11,159 (55.8%)
Charlson comorbidity index (CCI)*	
0	15,159 (75.8%)
1	2,802 (14.0%)
2	1,131 (5.7%)
3 to 12	904 (4.5%)

*CCI is derived from AHD

Table 2. Morbidity frequencies and measures of agreement by diagnosis, (N=19,996), CARTaGENE

Diseases	Concordant cases*	Frequency (%)		Sensitivity (%) (95% CI)	Specificity (%) (95% CI)	PPV (%) (95% CI)	Kappa (95% CI)
		CaG	AHD				
Chronic diseases							
Hypertension	3,660	25.00	22.80	73.83 (72.15-75.51)	94.15 (92.25-96.05)	80.79 (79.03-82.55)	0.70 (0.69-0.71)
OA	1,554	16.10	20.50	38.48 (37.26-39.70)	89.70 (87.84-91.56)	49.05 (47.68-50.42)	0.31 (0.29-0.32)
Depression	1,717	16.70	18.40	51.16 (49.76-52.56)	91.59 (89.71-93.47)	57.83 (56.34-59.32)	0.45 (0.43-0.47)
Diabetes	1,338	7.60	8.00	85.06 (83.25-86.87)	99.15 (97.2-100)	89.74 (87.88-91.6)	0.86 (0.85-0.88)
COPD	340	5.69	7.62	22.46 (21.53-23.39)	95.69 (93.77-97.61)	30.06 (28.99-31.13)	0.21 (0.18-0.23)
Hypothyroidism	932	7.10	7.60	61.52 (59.98-63.06)	97.4 (95.47-99.33)	66.1 (64.51-67.69)	0.61 (0.59-0.63)
Asthma	991	13.00	6.40	78.15 (76.42-79.88)	91.43 (89.56-93.3)	38.31 (37.1-39.52)	0.47 (0.45-0.49)
Chronic renal failure	44	0.31	3.30	5.09 (4.65-5.53)	99.91 (97.95-100)	72.13 (70.47-73.79)	0.09 (0.07-0.12)
Stroke	170	1.65	2.06	41.36 (40.10-42.62)	99.18 (97.23-100)	51.67 (50.26-53.08)	0.45 (0.40-0.49)
IBS	170	4.48	1.82	46.96 (45.62-48.3)	96.31 (94.39-98.23)	19.10 (18.24-19.96)	0.25 (0.22-0.29)
MI	296	2.81	1.68	88.62 (86.77-90.47)	98.66 (96.71-100)	52.95 (51.52-54.38)	0.66 (0.62-0.69)
Crohn's disease	103	0.60	1.40	38.01 (36.80-39.22)	99.91 (97.95-100)	85.83 (84.01-87.65)	0.52 (0.46-0.58)
RA	123	2.90	0.88	71.10 (69.45-72.75)	97.74 (95.8-99.68)	21.81 (20.89-22.73)	0.33 (0.28-0.37)
Schizophrenia	58	0.38	0.82	35.37 (34.20-36.54)	99.91 (97.95-100)	77.33 (75.61-79.05)	0.48 (0.41-0.56)
MS	74	0.41	0.66	56.49 (55.02-57.96)	99.96 (98-100)	90.24 (88.38-92.1)	0.69 (0.62-0.76)
Epilepsy	65	0.56	0.55	59.63 (58.12-61.14)	99.76 (97.80-100)	58.04 (56.55-59.53)	0.59 (0.51-0.66)
Cirrhosis	26	0.28	0.41	31.71 (30.61-32.81)	99.85 (97.89-100)	47.27 (45.92-48.62)	0.38 (0.27-0.48)
Parkinson’s disease	24	0.12	0.32	38.10 (36.89-39.31)	100 (98.04-100)	100 (98.04-100)	0.55 (0.43-0.68)
SLE	23	0.28	0.32	35.94 (34.76-37.12)	99.84 (97.88-100)	41.82 (40.55-43.09)	0.39 (0.27-0.5)
Cancers							
Breast	327	1.76	1.90	86.28 (84.46-88.1)	99.88 (97.92-100)	93.43 (91.54-95.32)	0.90 (0.87-0.92)
Prostate	169	0.89	1.26	67.6 (65.99-69.21)	99.95 (97.99-100)	94.94 (93.03-96.85)	0.79 (0.75-0.83)
Melanoma	72	1.67	0.80	45.28 (43.96-46.6)	98.68 (96.73-100)	21.62 (20.71-22.53)	0.29 (0.23-0.34)

Colon	61	0.42	0.70	43.57 (42.28-44.86)	99.89 (97.93-100)	73.49 (71.81-75.17)	0.55 (0.47-0.62)
Non-Hodgkin's lymphoma	39	0.22	0.53	36.79 (35.6-37.98)	99.97 (98.01-100)	88.64 (86.79-90.49)	0.52 (0.42-0.62)
Lung	45	0.27	0.48	47.37 (46.02-48.72)	99.96 (98-100)	84.91 (83.1-86.72)	0.61 (0.51-0.7)
Cervical	21	0.66	0.38	28 (26.96-29.04)	99.45 (97.5-100)	16.03 (15.25-16.81)	0.20 (0.13-0.27)
Bladder	40	0.22	0.34	58.82 (57.32-60.32)	99.98 (98.02-100)	93.02 (91.13-94.91)	0.72 (0.63-0.82)
Thyroid	43	0.27	0.30	71.67 (70.01-73.33)	99.94 (97.98-100)	79.63 (77.88-81.38)	0.75 (0.67-0.84)
Kidney	31	0.17	0.27	57.41 (55.92-58.9)	99.98 (98.02-100)	91.18 (89.31-93.05)	0.70 (0.6-0.81)
Rectum	12	0.08	0.25	24 (23.04-24.96)	99.98 (98.02-100)	75 (73.30-76.70)	0.36 (0.21-0.51)
Uterus	18	0.39	0.19	47.37 (46.02-48.72)	99.7 (97.74-100)	23.08 (22.14-24.02)	0.31 (0.20-0.42)

* Concordant cases represent the double positive for self-reported CaG diagnosis and AHD (consult text for specific definition).

CaG: Self-reported diagnosis in the CARTaGENE cohort; ADH: Administrative health data; CI: Confidence Interval; COPD: Chronic obstructive pulmonary disease; IBS: Irritable bowel syndrome; MS: Multiple sclerosis; MI: Myocardial infarction; OA: Osteoarthritis; RA: Rheumatoid arthritis; SLE: Systemic lupus erythematosus.

Table 3. Fit statistics for the complete logistic models where concordant cases were higher than 30, CARTaGENE

Diseases	Pseudo-R^2	Model Wald p	Sex p	Age groups p	Regions p	Education p	Income quintiles p	Heavy health utilization p	CCI* p
Chronic diseases									
Hypertension	0.0246	<0.0001	<0.0001	<0.0001	0.0254	0.1090	<0.0001	<0.0001	<0.0001
OA	0.0561	<0.0001	<0.0001	<0.0001	0.001	0.0033	0.0003	<0.0001	0.13
Depression	0.0282	<0.0001	<0.0001	0.0004	0.0002	0.06	<0.0001	<0.0001	0.86
Diabetes	0.0432	<0.0001	<0.0001	<0.0001	0.3250	0.3736	0.3928	0.0003	<0.0001
COPD	0.0358	<0.0001	<0.0001	0.64	0.0031	<0.0001	<0.0001	<0.0001	<0.0001
Hypothyroidism	0.0572	<0.0001	<0.0001	<0.0001	0.33	0.72	0.34	<0.0001	0.18
Asthma	0.0196	<0.0001	<0.0001	<0.0001	0.0003	0.79	<0.0001	<0.0001	<0.0001
Chronic renal failure	0.0649	<0.0001	<0.0001	0.0236	0.0216	0.0410	0.0120	<0.0001	<0.0001
Stroke	0.0679	<0.0001	0.0114	<0.0001	0.07	0.81	0.0146	<0.0001	<0.0001
IBS	0.0439	<0.0001	<0.0001	0.09	0.27	0.41	0.17	<0.0001	0.82
MI	0.1278	<0.0001	<0.0001	<0.0001	0.78	0.41	0.0063	<0.0001	<0.0001
Crohn's disease	0.0305	<0.0001	0.22	0.77	0.37	0.93	0.48	<0.0001	0.0230
RA	0.0497	<0.0001	0.08	<0.0001	0.0017	0.0452	<0.0001	<0.0001	0.14
Schizophrenia	0.1158	<0.0001	0.0173	0.0043	0.21	0.91	<0.0001	<0.0001	0.23
MS	0.0290	0.0628	0.06	0.08	0.23	0.45	0.25	0.053	0.80
Epilepsy	0.0300	0.0023	0.90	0.76	0.61	0.73	0.43	0.0042	0.0082
Cancers									
Breast	0.1026	<0.0001	<0.0001	0.63	0.77	0.40	0.54	0.0090	<0.0001
Prostate	0.2108	<0.0001	NA	<0.0001	0.0389	0.15	0.71	0.0150	<0.0001
Melanoma	0.0393	<0.0001	0.49	<0.0001	0.14	0.54	0.0047	<0.0001	<0.0001
Colon	0.1251	<0.0001	0.0017	0.09	0.72	0.17	0.18	<0.0001	<0.0001
Non-Hodgkin's lymphoma	0.1631	<0.0001	0.0152	0.91	0.32	0.08	0.15	0.0002	<0.0001
Lung	0.1134	<0.0001	0.13	0.21	0.0089	0.34	0.39	0.0013	<0.0001
Bladder	0.0923	<0.0001	0.73	0.06	0.98	0.93	0.84	0.14	<0.0001
Thyroid	0.1140	0.0001	0.32	0.23	0.84	0.13	0.41	0.0074	<0.0001
Kidney	0.0998	<0.0001	0.92	0.26	0.78	0.051	0.53	0.06	<0.0001

p: probabilities; COPD: Chronic obstructive pulmonary disease; IBS: Irritable bowel syndrome; MS: Multiple sclerosis; MI: Myocardial infarction; OA: Osteoarthritis; RA: Rheumatoid arthritis; SLE: Systemic lupus erythematosus; CCI: Charlson comorbidity index; NA: no model possible.

Fig. 1 Selected co-factor effects of agreement between diseases.

A) Sex effect: women versus men

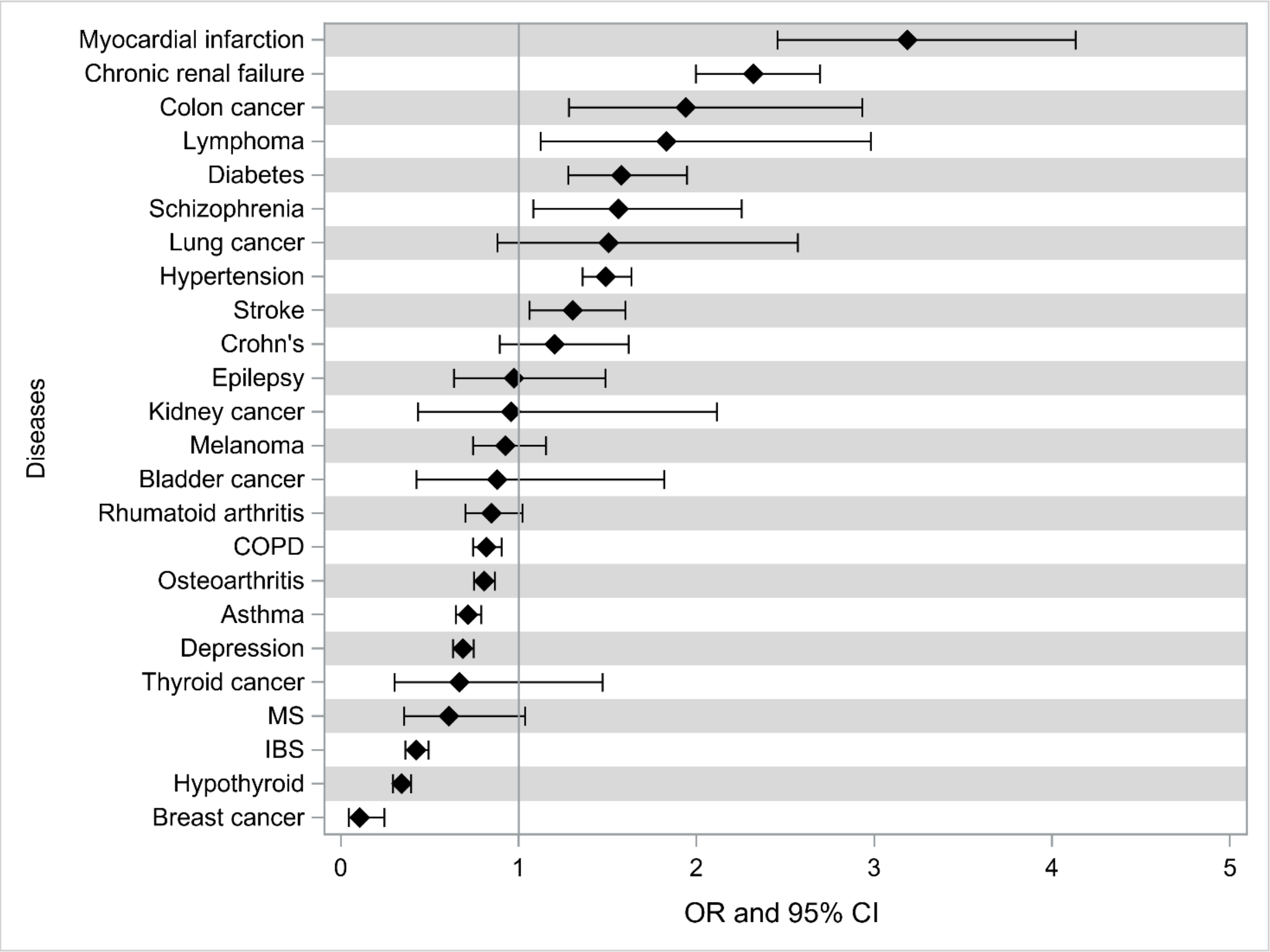

OR greater than 1 means that agreement is stronger in women compared to men.
COPD: Chronic obstructive pulmonary disease; IBS: Irritable bowel syndrome; MS: Multiple sclerosis; Lymphoma: Non Hodgkin’s lymphoma; SLE: Systemic lupus erythematosus.

B) Health care utilization effect: heavy vs. light users

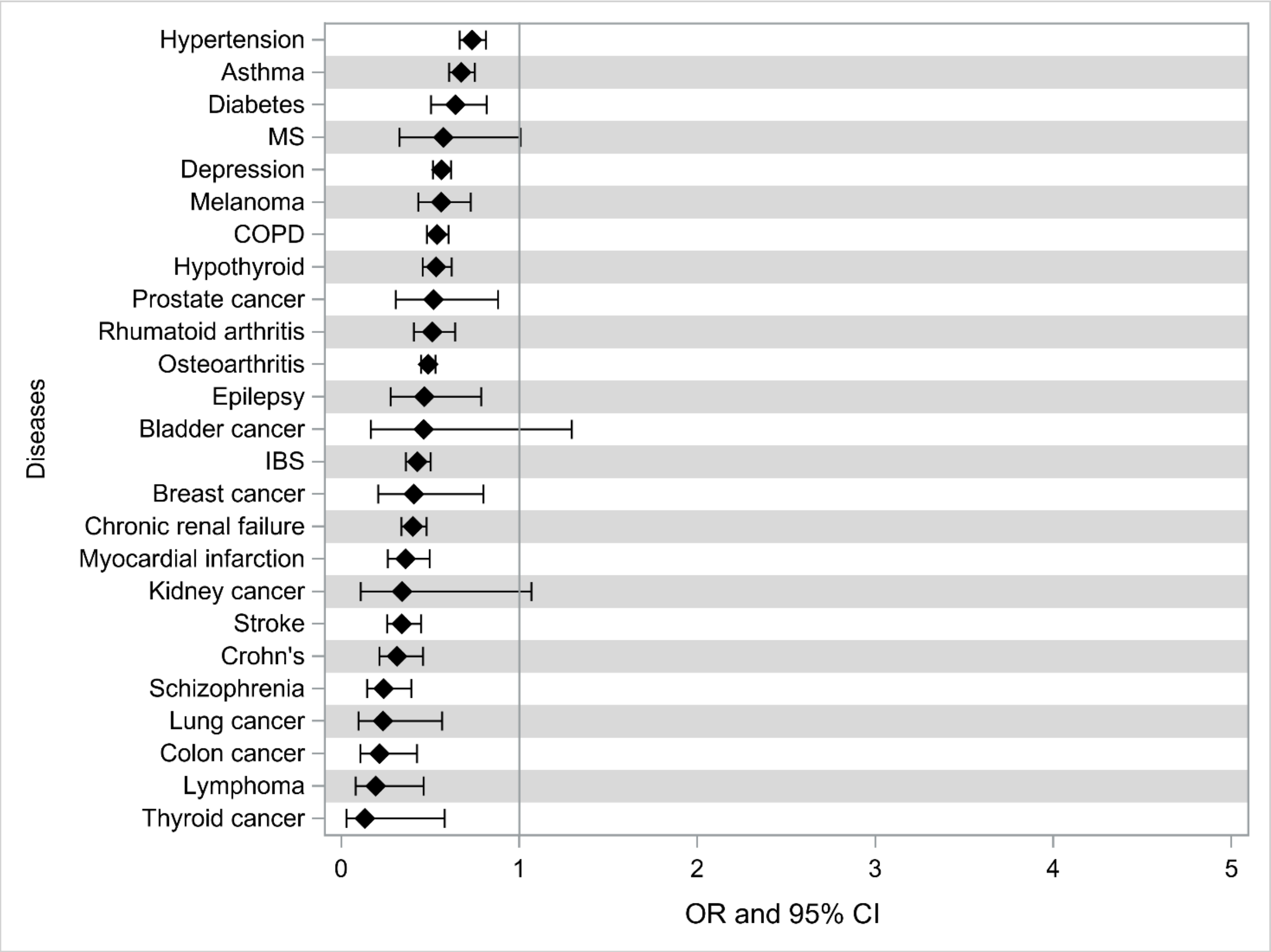

OR greater than 1 means that agreement is stronger in heavy health care users compared to light users. COPD: Chronic obstructive pulmonary disease; IBS: Irritable bowel syndrome; MS: Multiple sclerosis; Lymphoma: Non Hodgkin's lymphoma; SLE: Systemic lupus erythematosus.

C) Comorbidity effect: increase in one unit of the Charlson comorbidity index

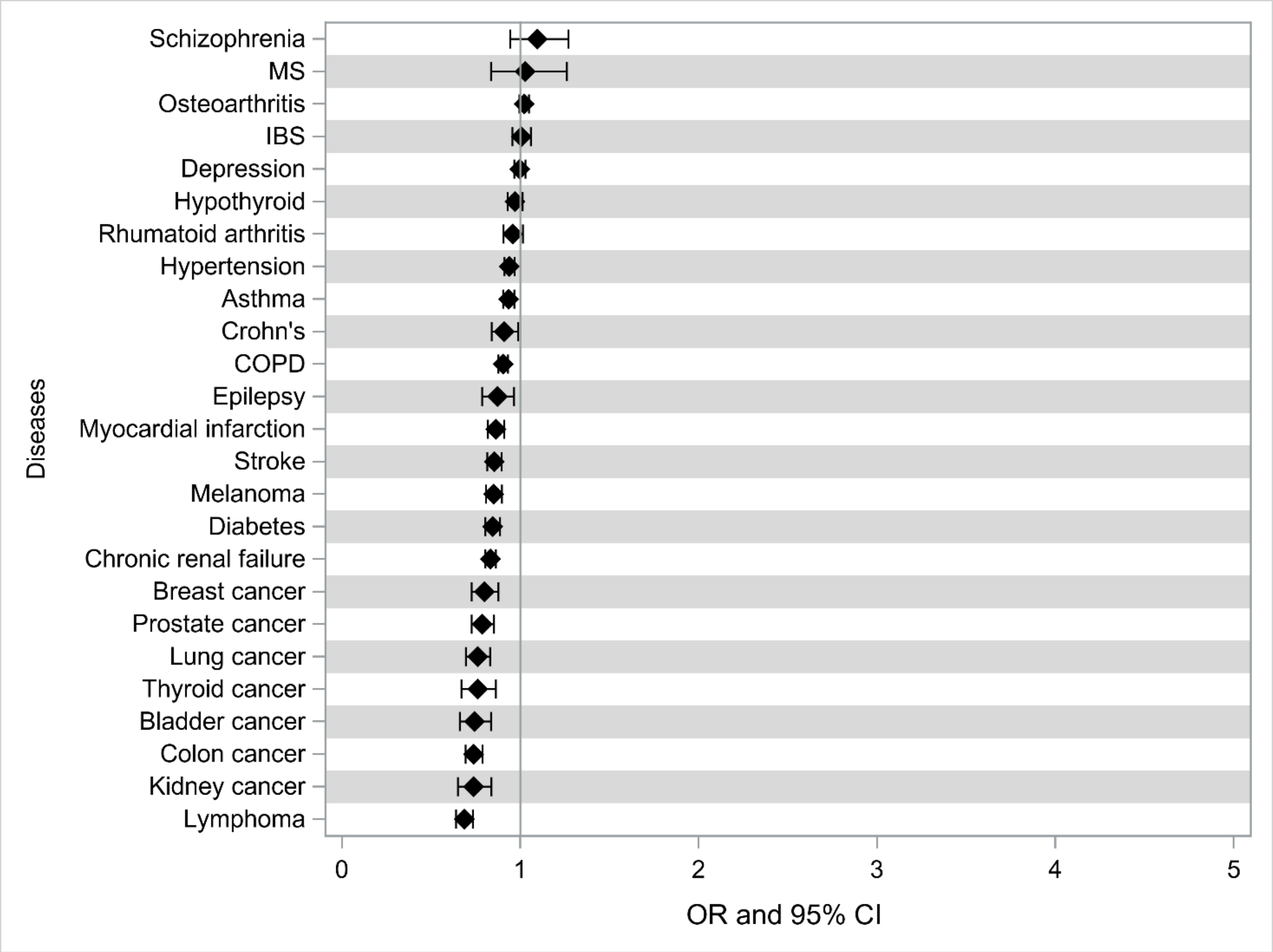

OR greater than 1 means that agreement is stronger for each unit increase of the Charlson comorbidity index.
COPD: Chronic obstructive pulmonary disease; IBS: Irritable bowel syndrome; MS: Multiple sclerosis; Lymphoma: Non Hodgkin’s lymphoma; SLE: Systemic lupus erythematosus.

D) Older age effect: 60-69 years old versus 40-49 years old

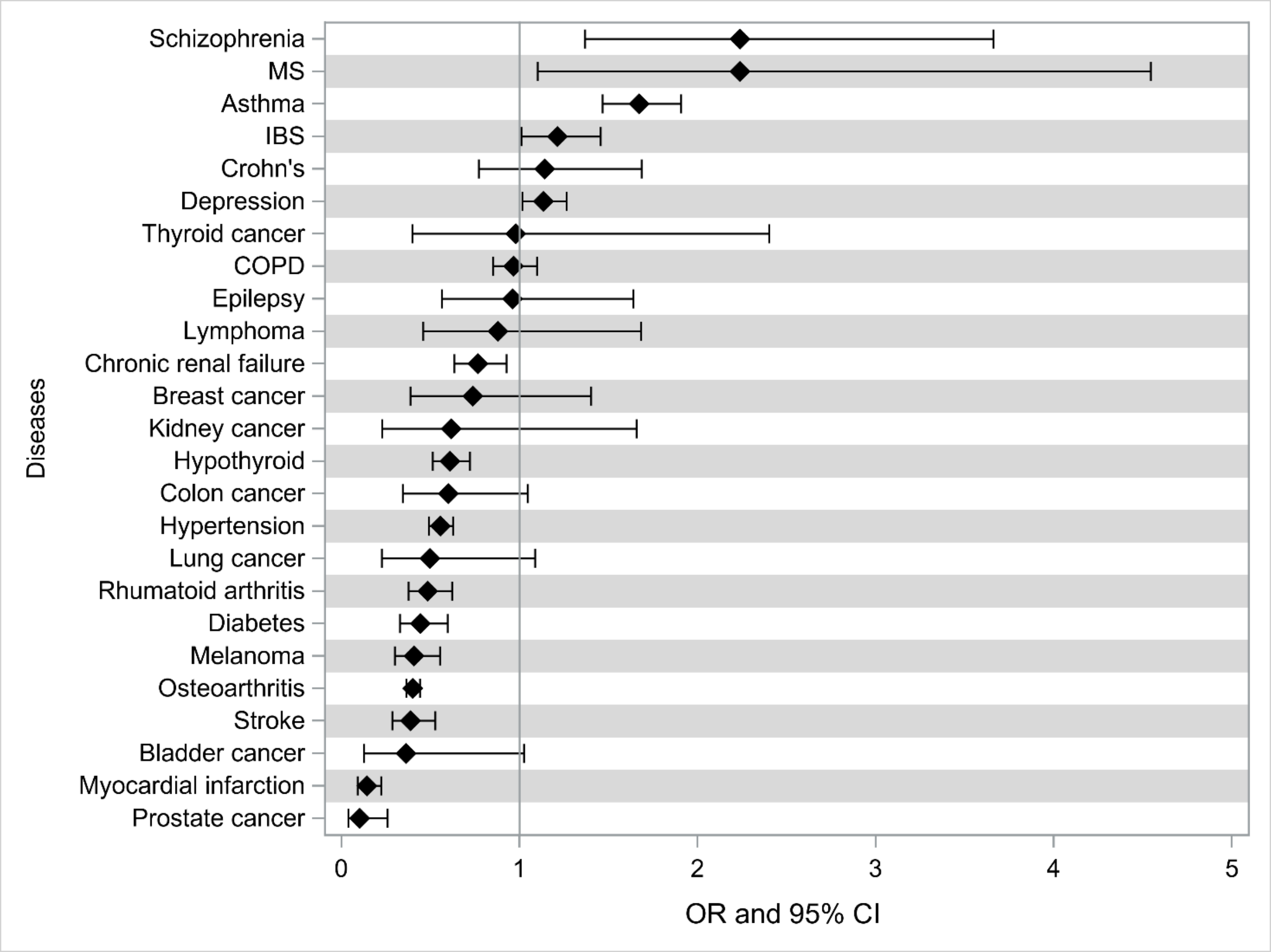

OR greater than 1 means that agreement is stronger in older participants compared to the younger ones. COPD: Chronic obstructive pulmonary disease; IBS: Irritable bowel syndrome; MS: Multiple sclerosis; Lymphoma: Non Hodgkin’s lymphoma; SLE: Systemic lupus erythematosus.

E) Income effect: highest versus lowest income quintiles

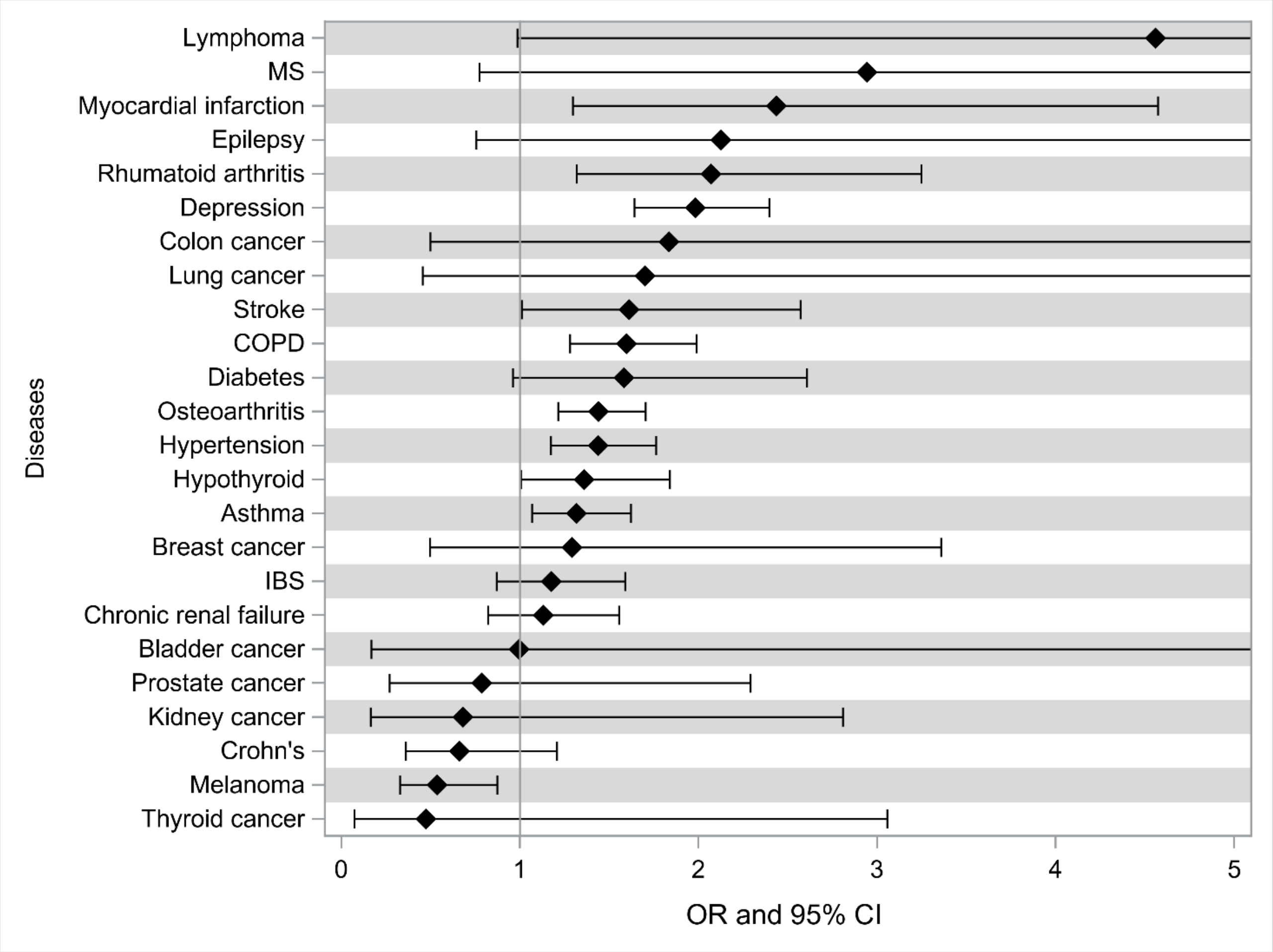

OR greater than 1 means that agreement is stronger in the highest quintile group compared to the lowest. COPD: Chronic obstructive pulmonary disease; IBS: Irritable bowel syndrome; MS: Multiple sclerosis; Lymphoma: Non Hodgkin's lymphoma; SLE: Systemic lupus erythematosus.

APPENDIX A -List of selected conditions/morbidities, the self-reported survey questions used to access them, as well as the specific algorithm and ICD codes used on MED-ÉCHO administrative health data [a].

Disease	Self-reported survey question	Administrative health database algorithm	ICD-9 relevant codes	ICD-10 relevant codes
Asthma	Has a doctor ever told you that you had asthma?	1 hospitalization or 3 physician claims in 2 years or less	493	J45
Chronic renal failure	Has a doctor ever told you that had kidney disease such as renal failure, renal infection or a kidney stones?<br>What type of renal failure was it?	1 hospitalization or 3 claims in 1 year	583, 584, 585, 586, 592, 593.9	N00-N23
Cirrhosis[b]	Has a doctor ever told you that you had a liver disorder such as cirrhosis or chronic hepatitis?<br>What kind of liver disorder was it?	1 hospitalization or 1 physician claim	571.2, 571.5, 571.6	K70.3, K74.3 K74.4, K74.5, K74.6
Chronic obstructive pulmonary disease (COPD)[c]	Has a doctor ever told you that you had chronic bronchitis?	1 hospitalization or 2 physician claims in 2 years or less	416.8, 416.9, 490–92, 494-505, 506.4, 508.1, 508.8	I27.8, I27.9, J40–J44, J46-J47, J60–J67, J68.4, J70.1, J70.3
Crohn's disease[d]	Has a doctor ever told you that you suffer from a bowel disorder such as Crohn's Disease, Ulcerative colitis, Irritable bowel syndrome, polyps or diverticular disease?<br>What kind of bowel disorder was it?	2 hospitalizations or 2 physician claims in 3 years or less	555	K50
Depression	Has a doctor ever told you that you had depression?	1 hospitalization or 2 physician claims in 2 years or less	296.2, 296.3, 296.5, 300.4, 309, 311	F20.4, F31.3–F31.5, F32, F33, F34.1, F41.2, F43.2
Diabetes	Has a doctor ever told you that you had diabetes?	1 hospitalization or 2 physician claims in 3 years or less, exclude gestational diabetes	250	E10-E14
Epilepsy	Has a doctor ever told you that you had a neurological condition or migraines?<br>What type of neurological condition was this?	1 hospitalization or 2 physician claims in 2 years or less	345	G40-G41

Hypertension	Has a doctor ever told you that you had high blood pressure or hypertension, other than high blood pressure occurring only in pregnancy?	1 hospitalization or 2 physician claims in 3 years or less, exclude gestational hypertension	401-405	I10-I13, I15
Hypothyroidism	Has a doctor ever told you that you had thyroid disease?<br>Is (or was) it hyperthyroidism or hypothyroidism?	1 hospitalization or 2 physician claims in 2 years or less	240.9, 243, 244, 246.1, 246.8	E00–E03, E89.0
Irritable bowel syndrome (IBS)	Has a doctor ever told you that you suffer from a bowel disorder such as Crohn's Disease, Ulcerative colitis, Irritable bowel syndrome, polyps or diverticular disease?<br>What kind of bowel disorder was it?	1 hospitalization or 2 physician claims in 2 years or less	564.1	K58
Multiple sclerosis (MS)	Has a doctor ever told you that you had a neurological condition or migraines?<br>What type of neurological condition was this?	2 hospitalizations or 2 physician claims in 3 years or less	323, 340, 341.0, 341.9, 377.3	G35, G36, G37, H46
Myocardial infarction (MI)	Has a doctor ever told you that you have had a myocardial infarction or a heart attack?	1 hospitalization	410	I21-I22
Osteoarthritis (OA)	Has a doctor ever told you that you had arthritis?<br>What kind of arthritis is (or was) it?	1 hospitalization or 1 physician claim in 2 years or less	715	M15-M19
Parkinson's disease	Has a doctor ever told you that you had a neurological condition or migraines?<br>What type of neurological condition was this?	1 hospitalization or 2 physician claims in 2 years or less	332	G20, G21, G22
Rheumatoid arthritis (RA)	Has a doctor ever told you that you had arthritis?<br>What kind of arthritis is (or was) it?	1 hospitalization or 3 physician claims, one by rheumatologist, in 2 years or less	714	M05-M06
Schizophrenia	Has a doctor ever told you that you had Schizophrenia?	1 hospitalization or 2 physician claims in 2 years or less	295	F20, F21, F23.2, F25
Systemic lupus erythematosus (SLE)	Has a doctor ever told you that you suffer from systemic lupus erythematosus, often called<br>SLE or "Lupus"?	1 hospitalization or 3 physician claims, one by rheumatologist, in 2 years or less	710	M32

Stroke	Has a doctor ever told you that you have had a stroke?	1 hospitalization or 1 physician claim	430, 431, 433.x1, 434.x1, 435,436	I60, I61, I63, I64
Bladder cancer	Has a doctor ever told you that you had cancer or a malignancy of any kind? What type of cancer or malignancy of any kind was it?	1 hospitalization or 2 physician claims in 2 years or less	188, 233.7	C67, D09.0
Breast cancer	Has a doctor ever told you that you had cancer or a malignancy of any kind? What type of cancer or malignancy of any kind was it?	1 hospitalization or 2 physician claims in 2 years or less	174, 233.0	C50, D05
Cervical cancer	Has a doctor ever told you that you had cancer or a malignancy of any kind? What type of cancer or malignancy of any kind was it?	1 hospitalization or 2 physician claims in 2 years or less	180, 233.1	C53 D06
Uterine cancer	Has a doctor ever told you that you had cancer or a malignancy of any kind? What type of cancer or malignancy of any kind was it?	1 hospitalization or 2 physician claims in 2 years or less	182, 233.2	C55
Prostate cancer	Has a doctor ever told you that you had cancer or a malignancy of any kind? What type of cancer or malignancy of any kind was it?	1 hospitalization or 2 physician claims in 2 years or less	185, 233.4	C61, D07.5
Colon cancer	Has a doctor ever told you that you had cancer or a malignancy of any kind? What type of cancer or malignancy of any kind was it?	1 hospitalization or 2 physician claims in 2 years or less	153, 230.3	C18
Rectum cancer	Has a doctor ever told you that you had cancer or a malignancy of any kind? What type of cancer or malignancy of any kind was it?	1 hospitalization or 2 physician claims in 2 years or less	154, 230.4-230.6	C19-C21
Kidney cancer	Has a doctor ever told you that you had cancer or a malignancy of any kind? What type of cancer or malignancy of any kind was it?	1 hospitalization or 2 physician claims in 2 years or less	189.0	C64

Lung cancer	Has a doctor ever told you that you had cancer or a malignancy of any kind? What type of cancer or malignancy of any kind was it?	1 hospitalization or 2 physician claims in 2 years or less	C34, C38.4	162.2-162.9 163 231.2
Trachea cancer	Has a doctor ever told you that you had cancer or a malignancy of any kind? What type of cancer or malignancy of any kind was it?	1 hospitalization or 2 physician claims in 2 years or less	162.0	C33
Non-Hodgkin's lymphoma	Has a doctor ever told you that you had cancer or a malignancy of any kind? What type of cancer or malignancy of any kind was it?	1 hospitalization or 2 physician claims in 2 years or less	200, 202, 203.0, 238.6	C82–C85, C88, C90.0, C90.2, C96
Melanoma	Has a doctor ever told you that you had cancer or a malignancy of any kind? What type of cancer or malignancy of any kind was it?	1 hospitalization or 2 physician claims in 2 years or less	172, 232	C43, D03
Thyroid cancer	Has a doctor ever told you that you had cancer or a malignancy of any kind? What type of cancer or malignancy of any kind was it?	1 hospitalization or 2 physician claims in 2 years or less	193	C73, D09.3

[a] Adapted from Quan *et al.* (2005)[34] and Tonelli *et al.* (2015)[35]
[b] Includes alcohol misuse and cirrhosis (571.2, K70.3).
[c] Combination of chronic bronchitis and emphysema.
[d] Original algorithm also includes other type inflammatory bowel disease. ICD codes were restricted to Crohn's disease
